# How to RESPOND to Modern Challenges for People Living with HIV: A Profile for a New Cohort Consortium

**DOI:** 10.3390/microorganisms8081164

**Published:** 2020-07-31

**Authors:** The RESPOND Study Group

**Keywords:** HIV, cohort, observational study, hepatitis, public health, tuberculosis, pharmacovigilance

## Abstract

Background: the International Cohort Consortium of Infectious Disease (RESPOND) is a collaboration dedicated to research on HIV and other infectious diseases. Methods: RESPOND is a flexible organization, with several independent substudies operating under one shared governance. HIV-related variables, including full antiretroviral therapy (ART) history, are collected annually for all participants and merged with substudy specific data into a shared data pool. Incident clinical events are reported using standardized forms. Prospective follow-up started 1/10/17 (enrolment) with retrospective data collected back to 01/01/12. Results: Overall, 17 cohorts from Europe and Australia provided data on 26,258 people living with HIV (PLWH). The majority (43.3%) of the population were white, with men-sex-with-men accounting for 43.3% of the risk for HIV acquisition. The median age was 48 years (IQR 40–56) and 5.2% and 25.5% were known to be co-infected with hepatitis B or C. While 5.3% were ART-naïve, the median duration on ART was 10.1 years (4.8–17.6), with 89.5% having a VL &lt;200 copies/mL and the median CD4 count being 621 cells/µL (438–830). Malignancies (*n* = 361) and cardiovascular disease (*n* = 168) were the predominant reported clinical events. Conclusion: RESPOND’s large, diverse study population and standardized clinical endpoints puts the consortium in a unique position to respond to the diverse modern challenges for PLWH.

## 1. Introduction

The International Cohort Consortium of Infectious Disease (RESPOND) was formed in 2017 as a prospective, multi-cohort collaboration for the study of infectious diseases, with a special focus on people living with HIV (PLWH). RESPOND was founded upon the groundwork laid by outstanding European HIV cohort collaborations such as EuroSIDA, the Collaboration of Observational HIV Epidemiological Research Europe (COHERE), and Data Collection on Adverse events of Anti-HIV Drugs (D:A:D) studies, and utilizes a similar, well-established infrastructure [1,2,3,4].

RESPOND offers a research framework with a flexible organization, applying a common data model across different substudies, utilizing one shared data pool. Additionally, all involved in RESPOND can contribute to the ongoing scientific agendas. Together, these dynamic features facilitate responses to a broad range of unmet research needs.

The large size of RESPOND allows scientific questions to be addressed that individual cohorts do not have the size to investigate themselves. Furthermore, the heterogeneous study population with participants from across the whole of Europe and Australia and a high degree of data quality—including the central validation of standardized clinical event definitions—ensure that the results are reliable and applicable to a broader population of PLWH. RESPOND will address clinically relevant research questions, such as the risk and outcomes of non-AIDS comorbidities and the possible relationship to long-term ART exposure; to the outcomes and treatment of viral hepatitis B (HBV), hepatitis C (HCV), and tuberculosis (TB) co-infections; and to support public health initiatives.

In this cohort profile, we will detail RESPOND’s organization, substudies, data collection and quality assurances, baseline characteristics, scientific findings to date, and focus areas going forward.

## 2. Materials and Methods:

### 2.1. Cohort Participation

The cohorts that constitute RESPOND are largely collaborators from the EuroSIDA-, COHERE-, and D:A:D multi-cohort studies. However, the consortium is an open network that welcomes additional cohorts to join if predefined criteria regarding the inclusion of a minimum number of participants, the need for a designated data manager, and data quantity- and quality are met. The first (and current) version of these criteria can be found in full at https://chip.dk/Studies/RESPOND/Study-documents [5].

### 2.2. Inclusion Criteria

**Figure 1:**
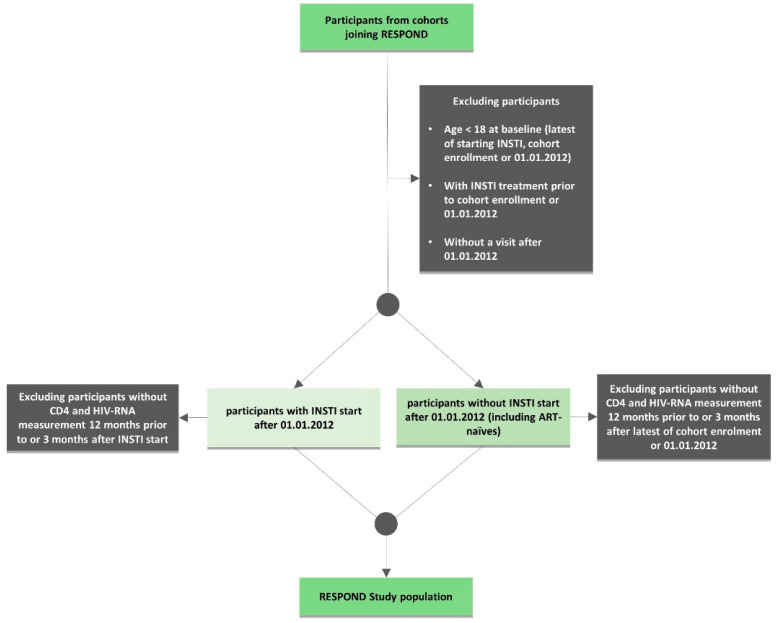
Flowchart of the International Cohort Consortium of Infectious Disease (RESPOND) inclusion and exclusion criteria.

PLWH older than 18 years were eligible for inclusion. Each cohort contributed a pre-defined minimum number of participants related to the size of the specific cohort, according to agreed inclusion and exclusion criteria carefully designed to include a representative population. As RESPOND focuses on contemporary outcomes in PLWH, only individuals under prospective follow-up after 2011 were included. Two groups of individuals were enrolled; the first group started an integrase strand transfer inhibitor (INSTI) after 1/1/2012, with the baseline defined as the date of starting the INSTI. The second group did not start an INSTI at enrolment and had baseline defined as the latest of 1/1/2012 or enrolment into the local cohort. The participants from both groups were required to have a CD4 count and HIV viral load (VL) measurement in the 12 months prior to or within 3 months after baseline (Figure 1) [6].

To minimize the risks of selection and survival bias to the greatest extent possible, individuals who had died or had been lost to follow up in the period 1/1/2012–1/10/2017 were enrolled for the initial merger (more details in Section 2.6 on data collection and quality assurance).

### 2.3. Ethical Considerations

Studies performed within the RESPOND consortium are conducted according to the Declaration of Helsinki [7] and the requirements of Good Clinical Practice (GCP), as defined in the European Union’s (EU) GCP Directive [8]. All the data supplied to RESPOND follow local or national guidelines as appropriate, and enrolled participants are pseudonymized by the assignment of a unique identifier by the participating cohort before data transfer. As data controller, the Coordinating Centre (CC) located within the Danish Capital Region of Copenhagen, Denmark, stores, shares, and protects data in accordance with current legislation and under approval by The Danish Data Protection Agency (j.nr.: RH-2018-15, 26/1/2018), currently under the EU’s General Data Protection Regulation (EU) 2016/679.

RESPOND is registered at Clinicaltrial.gov (identifier: NCT04090151, 16/9/2019).

### 2.4. Consortium Organization and Governance:

**Figure 2:**
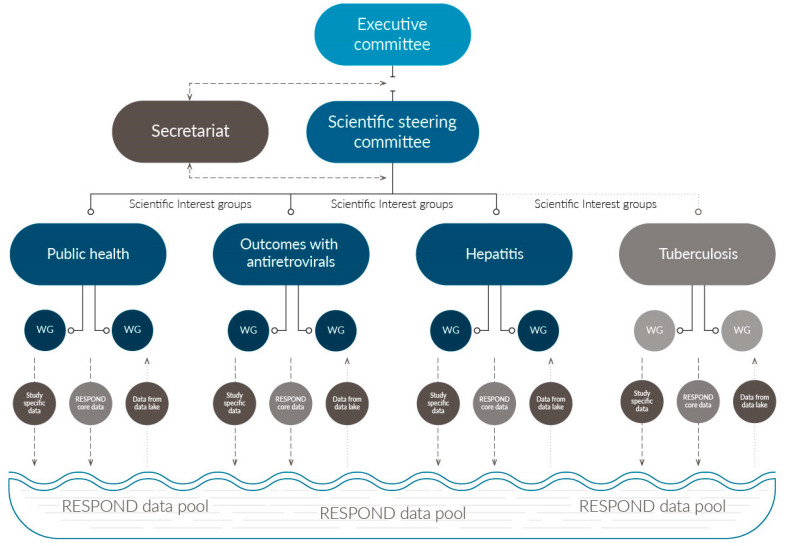
Organization of RESPOND. The executive committee oversee the overall financial wellbeing and scientific progression, while the scientific steering committee supervises the scientific integrity and research conducted. Research agendas are developed in the sub studies/scientific interest groups: Public Health, Outcomes with Antiretroviral Treatment, Hepatitis and Tuberculosis (the latter under development). Each SIG may have multiple underlying working groups (WG) dedicated to developing specific subtopics. All the participating cohorts supply core RESPOND data to the data pool. In addition, study-specific data can be collected by a subset of cohorts involved in specific SIGs which are also merged into the data pool. All the data can be utilized by the different SIGs upon permission from the cohorts.

The consortium is overseen by an Executive Committee (EC), which is composed of scientific stakeholders, representatives from the three largest participating cohorts, and the chairs from Scientific Steering Committee (SSC) (Figure 2). The role of the EC is to ensure the financial and structural integrity of RESPOND and to safeguard that study objectives are being met in a timely manner. However, the EC has no direct involvement in formulating specific scientific agendas, which is carried out under the supervision of the SSC [6]. The principal investigators from all the participating cohorts; HIV community representatives; research moderators; and scientific stakeholders, recruited based on their academic credentials, form the RESPOND SSC [6].

The scientific agendas that drive the consortium’s scientific progression are formed under several substudies termed scientific interest groups (SIGs; Figure 2). These substudies utilize the common RESPOND data pool and have the option to collect additional substudy specific data. This flexible and open structure enables new substudies to easy be added to the consortium’s on-going operations if imminent research questions arise. Participation in the SIGs is open for all individuals associated with RESPOND cohorts, community, funders, and external experts.

The RESPOND SIGs are formed and managed by two scientific moderators that are responsible for organizing all scientific activities within the SIG and for ensuring a contemporary research agenda. Relevant variables for SIG analyses may or may not already be collected as part of the standard RESPOND data collection. If a collection of new variables is needed, funds for the collection of these and following analysis must first be obtained, and this relies on the SIG participants and moderators. Therefore, the time from conceptualizing to a functioning SIG largely depends on the SIG’s scientific focus and the availability of data. More information on the initiation of a SIG in RESPOND can be obtained via the RESPOND webpage (https://chip.dk/Studies/RESPOND) or by contacting the RESPOND secretariat (respond.rigshospitalet@regionh.dk).

Secretariat functions and data management are handled by the Coordinating Centre (CC). The statistical centers located at University College London, England, and Kirby Institute Sydney, Australia, manage data cleaning, statistical analyses, and support for cohort-led specific analyses.

### 2.5. Scientific Focus and Development of Scientific Agendas

At present, there are three operational SIGs: The Clinical Outcomes of Antiretroviral Treatment (Outcomes) SIG, the Hepatitis SIG, and the Public Health SIG. A TB SIG is under formation (Figure 2).

The Outcomes SIG investigates the incidence of, risk factors for, and outcomes related to non-AIDS comorbidities and the use of individual antiretroviral agents (ARVs). The Hepatitis SIG explores specific questions relevant for PLWH co-infected with HBV (with or without hepatitis D virus) and HCV. The purpose of the Public Health SIG is to implement studies of broader public health relevance using cohort clinical data in collaboration with EU surveillance institutions such as the European Center for Disease Control (ECDC), the World Health Organization (WHO), and other European-wide public health projects.

All the members of a SIG may propose scientific projects [9]. Two appointed moderators of the SIG will initially assess the overall relevance and feasibility, before the proposal is further discussed and developed within the wider SIG. Smaller working groups can be formed by SIG members with a special interest within the spectrum of an SIG’s research, taking advantage of the interest and expertise of other working group members (Figure 2).

### 2.6. Data Collection and Quality Assurance

The first data merger for RESPOND took place in 2017, collecting data for the period 01/01/12–01/10/2017. Since then, prospectively collected data from cohorts are annually submitted to the RESPOND CC and merged with the RESPOND data pool.

The clinical and demographic variables collected by RESPOND are summarized in Table 1. All the variables are categorized as either RESPOND core variables—which all participating cohorts are required to supply for all enrolled participants—or study-specific variables, which are only required for participants in specific substudies. Both types of variable are merged into the common data pool and can be included in all RESPOND substudies (Figure 2). Data can only be utilized upon approval by the individual cohorts that supplied the data, and participation in specific substudies can be retracted at any time, following the RESPOND governance [6].

RESPOND collects and stores data in the HIV Cohorts Data Exchange Protocol (HICDEP) format [10], detailed in the RESPOND Standard Operating Procedure [11]. Data is transferred to the RESPOND CC in one of two ways: by manually entering data via the secure, browser-based real-time Research Electronic Data Capture platform (REDCap) [12]; or by transferring larger amounts of data on designated templates, using the in-house developed RESPOND Electronic Submission Tool (REST). Both systems are operated from a secure location, managed by the Danish Capital Regions IT Department, and use secure communication (https), with only authorized personnel having access to the systems [13]. The submission of routine follow-up data occurs annually in the period between the 1st of October to the 1st of December.

Both REST and REDCap users will be met with multiple automated quality assurance checks when uploading or entering data. These built-in checks enable sites and cohorts to correct errors before data submission. Within a week of submission, raw datasets from individual cohorts are assessed for completeness and correctness by staff at the RESPOND CC. The cohorts are queried and asked to clarify and immediately resubmit corrected data as appropriate. After data cleaning, the statistical center produces comprehensive summaries of the data quality and completeness and provides individual feedback to cohorts, with the intent of improving data prior to the next data submission. In brief, assessments are made for all specific core variable since the last data submission. Correctness is assessed by a wide range of checks, aimed at identifying unintuitive reporting or systematic import errors (i.e., codes indicating a participant is receiving ≥2 INSTIs simultaneously, a participant who has no available HIV-RNA measurement available within the proximity of a change in ART, or a participant with no new serum creatinin measure).

Information on clinical events is collected using non-identifiable data on study-specific Case Report Forms (CRFs) for the following events: myocardial infarction (MI), stroke, invasive cardiovascular procedure (ICP; coronary angioplasties/stenting, coronary by-pass surgery, and carotid endarterectomy), end-stage liver and renal disease (ESLD and ESRD), and AIDS- and non-AIDS defining malignancy (ADM and NADM; excluding non-melanoma skin cancers and pre-cancers). Opposite from the routine follow-up data, CRFs are collected in real time from the period the 1st of December to the 1st of July. After CRFs are received at the CC, events are validated by a specially trained medical doctor following a prespecified algorithm [14], which mirrors the event definitions developed in the D:A:D study. To further increase the data quality, a proportion of all the validated events are reviewed by an external expert from the relevant medical specialty (i.e., MI events are reviewed by an external independent senior cardiologist). CRFs with causes of death (CoDe) must be submitted for all deceased participants, and the cause of death is coded by a trained medical doctor following the CoDe protocol [15].

Substantial quality assurance steps are in place to limit the potential underreporting of clinical events, including the assessment of the incidence rates of events reported from all individual cohorts. Likewise, the proportions of queried and dismissed events are continuously monitored to identify areas where individual cohorts may need to focus their attention and increase the amount and quality of collected data. Further, if a CRF does not contain the adequate information to allow for central validation, the cohorts are queried up to three times.

## 3. Results

### 3.1. Baseline Demographics and Clinical Caracteristics

By 1/10/17, 17 cohorts had agreed to engage in the consortium; 15 cohorts contributed to the first data merger, with a total of 26,258 individuals (Table 2). Two additional cohorts subsequently joined the second data merger in 2018. After the third data merger in 2019, RESPOND followed more than 34,000 PLWH.

At the study enrollment, the median age was 48 years (interquartile range, IQR, 40–56); 24.9% were younger than 40 years and 14.6% were older than 60 years. Women (25.7%) and persons of non-white ethnicity (27.6%) made up a substantial proportion of the study population. The RESPOND participants originated mainly from Western- (51.2%) and Southern Europe (26.6%), with 12.5% from Central Eastern- or Eastern Europe and 9.7% from Northern Europe (including Australia). Heterosexual contact and injecting drug use, respectively, accounted for 34.3% and 14.9% of the risk factors for HIV acquisition, with men-who-have-sex-with-men (MSM) being the largest group (43.4%). Hepatitis B- or C co-infection were seen in 5.2% and 25.5% of the participants. At the time of enrollment into RESPOND, the majority (89.5%) of participants had a VL <200 copies/mL and 67.0% had a CD4 count >500 cells/µL. The nadir CD4 count was ≤50 cells/µL for 16.4%. The median baseline date was 06/17 [12/15–09/17].

### 3.2. ART Exposure

**Figure 3:**
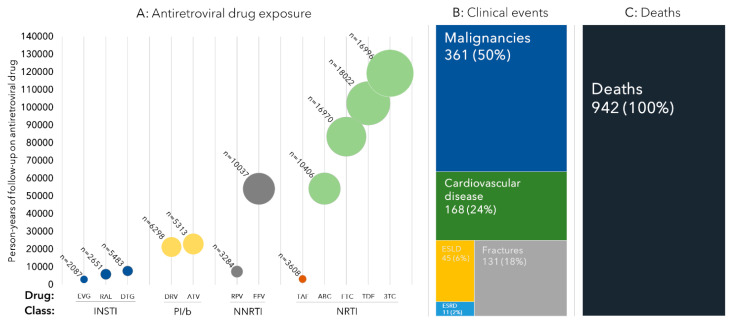
(**A**) Antiretroviral exposures at RESPOND enrolment; (**B**) number of validated clinical; (**C**) number of deaths in the study period events, after the 1st RESPOND data merger. Abbreviations: INSTI: Integrase strand transfer inhibitor; EVG/c: cobicistat boosted elvitegravir; RAL: Raltegravir; DTG: dolutegravir; PI/b: boosted protease inhibitor; DRV: darunavir; ATV: atazanavir; NNRTI: non-nucleotide reverse transcriptase inhibitor; RPV: rilpivirin; EFV: efavirenz; NRTI: nucleos(t)ide reverse transcriptase inhibitor; TAF: tenofovir alafenamide; ABC: abacavir; FTC: emtricitabine; TDF: tenofovir disoproxil; 3TC: lamivudine; ESLD: end-stage liver disease; ESRD: end-stage renal disease. Cardiovascular disease includes myocardial infarctions, strokes, and invasive cardiovascular procedures. Bubble size reflects the person-years of follow-up (PYFU) exposed to the specific antiretroviral drug, calculated from last clinical visit, with the number above each bubble indicating the number of individuals exposed. Clinical events refer to validated events after the first data merger. Deaths are all deaths occurring in the period between 01/01/12 and 01/10/2017.

While 5.3% were ART-naïve at enrollment, the median duration of ART was 10.1 years (4.8–17.6), (Figure 3a). Dolutegravir (DTG) was the INSTI with the greatest cumulative exposure time, followed by raltegravir (RAL) and cobicistat boosted elvitegravir (EVG/c) (7835, 6044, and 3100 person-years of follow-up (PYFU), respectively). For contemporary boosted protease inhibitors (PI/b), the duration of exposure to darunavir (DRV/b) and atazanavir (ATV/b) was relatively similar (21,400 and 23,060 PYFU). For non-nucleoside reverse transcriptase inhibitors (NNRTIs), the exposure time to efavirenz (EFV) was much higher than that to rilpivirine (RPV; 7521 and 54,236 PYFU). In descending order, the highest cumulative exposures for nucleot(s)ide-analogue reverse transcriptase inhibitors were seen for lamivudine (3TC; 119,388 PYFU), tenofovir disoproxil fumarate (TDF; 102,459 PYFU), emtricitabine (FTC; 83,613 PYFU), abacavir (ABC; 54,314 PYFU), and tenofovir alafenamide (TAF; 3294 PYFU).

### 3.3. Clinical Events

At time of the first data merger, the CC had received and centrally validated >90% of all expected event forms, with a total of 716 events. The predominant events were malignancies (*n* = 351 (50.4%), of which 35.9% were NADM and 14.5% were ADM; Figure 3b), followed by CVD (168 (23.5%), of which 9.4% were ICP, 7.4% were MI, and 6.7% stroke), fractures (131, 18.0%), ESLD (45, 6.0%), and ESRD (11, 2.0%). In addition, there were 942 deaths recorded in the period between 1/1/12 and 1/10/17.

### 3.4. First RESPOND Findings

The first analyses performed within the Outcomes SIG have focused on INSTI-based ART. Greenberg et al. [15] examined the uptake and discontinuation of RAL, EVG/c, and DTG among 9702 RESPOND participants. At 6 months after initiation, 9% had discontinued their INSTI treatment, with the main reason being related to drug toxicity (5%). Nervous system-related toxicities accounted for the highest proportion of toxicity-related discontinuations for DTG. The overall conclusion from the large and geographically diverse study setting was that even though specific adverse effects may be overrepresented for individual drugs, INSTIs were generally well tolerated by most participants.

In a second analysis by Neesgaard et al. [16], the virologic and immunologic outcomes of treatment with INSTIs were compared to the use of contemporary PI/b and NNRTIs in 13,703 participants, with a focus on subgroups stratified by ART experience and viremia at baseline. Using both a composite treatment outcome and on-treatment analysis, the 12 months durability and efficacy were evaluated. INSTI- and NNRTI-based regimens were consistently found to be more durable and efficacious than PI/b-based regimens. As a follow-up study, Mocroft et al. [17] explored possible differences in the virologic and immunologic outcomes for the subset of ART-naïves, depending on the age group (≤40, 40–50, and >50 years), CD4 count (≤350 versus >350 cells /mm^3^), or VL at ART initiation (≤100,000 versus >100,000 copies/mL), without finding strong evidence that the differences in treatment response were different in the key subgroups.

RESPOND have also focused analyses on the contemporary question of ART strategies with two (2DR) or three drug (3DR) combinations. Greenberg et al. [18] compared the occurrence of severe clinical events among 1088 participants on 2DR to 8703 on 3DR. Persons on 2DRs tended to be older and have more comorbidities than those on 3DRs, and after accounting for these characteristics there was a similar short-term incidence of severe clinical events for those on 2DRs compared to those on 3DRs.

The Public Health SIG recently presented proof of concept data from a method of using data from a random sample of patients to estimate a clinic’s percentage of individuals virologically suppressed on ART to construct the right-hand side of the continuum of care in settings with fragmented data to support surveillance as well as quality control [19].

## 4. Discussion

RESPOND is a large multicenter cohort consortium with the participation of 17 large, pre-existing cohorts from across Europe and Australia. At present (2020), more than 34,000 PLWH have been enrolled. Building on the sound scientific collaborations ongoing for more than three decades in the EuroSIDA, COHERE, and D:A:D multicohort studies, RESPOND represents a flexible yet well-structured framework for addressing unmet scientific questions for HIV and other infectious diseases.

### 4.1. Pharmacovigilance and Comorbidities in a Modern Era

The development of well-tolerated, effective ART is one of the great milestones in modern medical history, having transformed HIV from a deadly progressive infection into a chronic condition for those with access to care [20]. However, lifelong treatment comes with a potential risk of cumulative long-term toxicities—and with the increased life expectancy [21], age-related comorbidities have become increasingly prevalent in PLWH [22,23,24,25]. Consequently, PLWH now need multi-disciplinary management, including the assessment of polypharmacy, potential drug-drug interactions, and the screening and management of comorbidities and co-infections.

Due to the large size, substantial heterogeneity, extended follow-up time, and systematic collection of centrally validated endpoints, RESPOND is in a unique position to conduct well-powered pharmacovigilance studies and to assess the impact of demographics, traditional risk factors, HIV-related factors, and ART on a wide range of comorbidities. RESPOND will systematically assess the safety of newer ARVs, with a particular focus on INSTIs and other ARVs that may still be used in only a subset of PLWH and that therefore require a large dataset to reliably assess the longer term outcomes. Furthermore, the large number of persons under follow-up will allow RESPOND to focus on well-defined clinical endpoints, where individual cohorts may have too limited statistical power to conduct reliable analyses. In addition, the large and diverse composition of the consortium population provides the possibility of subgroup analyses stratified by clinical characteristics such as sex, smoking status, and age, in addition to stratifications based on geographical- and economic regions. Thus, the results will be broadly generalizable to the population of PLWH seen in routine clinical care in across Europe and Australia.

Going forward, RESPOND will help generate pivotal information necessary for tailoring ART to meet the individual needs of an aging population of PLWH and to gain insights into the development and outcomes of comorbidities.

The RESPOND data pool already holds a significant amount of data on ARV exposures, comorbidities, and mortality, with the most common clinical events being CVD and malignancies. Therefore, RESPOND will focus on these clinical events in the shorter term. More specifically, analysis investigating the possible associations between cumulative INSTI exposure and the risk of CVD and malignancies are ongoing. Other analyses will investigate weight gain and dyslipidemia in PLWH using contemporary ART and causes of death in PLWH within RESPOND. Additional follow-up time will allow the analysis of other less frequently occurring comorbidities and the possible relation to the use and discontinuation of modern ART.

### 4.2. Hepatitis and Other Infectious Diseases

While HIV is the main focus area of RESPOND for the immediate future, there is a significant amount of research activity revolving around other co-infections within the consortium. Founded in the EuroSIDA cohort study network, the Hepatitis SIG has now been incorporated into the consortium and has recently added HCV treatment data and HBV-DNA to the already collected information on HCV-RNA and hepatitis serology. Among the ongoing research areas are the long-term outcomes of direct acting antivirals, HCV reinfection rates, and HBV/HDV co-infections. In addition, plans are being made to identify biomarkers predicting the development of hepatocellular carcinoma and investigations on the impact of non-alcoholic fatty liver disease and non-alcoholic steatohepatitis in PLWH.

TB is a leading cause of infection-related deaths worldwide [26], with high incidences in Sub-Saharan Africa, Asia, and Eastern Europe. It is also the most prevalent AIDS-defining disease and is overrepresented among PLWH [27]. Although TB was not part of the initial focus of RESPOND, the TB:HIV network [28] has now established links within RESPOND with the consortium’s dynamic framework, enabling the formation of a TB-focused substudy and the inclusion of basic TB-related variables into the latest RESPOND data collection. The SIG plans to examine the risk factors for TB, the use of newer antimycobacterial treatments, and the frequency of multiresistant TB strains, generating valuable knowledge about HIV/TB coinfections from a real-life perspective.

While these substudies do not provide an exhaustive list of co-infections for the population of PLWH, they show the ability of RESPOND to incorporate the standardized data collection of newly proposed data items across the network. As such, there is no limitation on which substudies can be undertaken within the consortium if there is a strong academic driving force and research need behind it.

### 4.3. Using Cohort Data to Support Public Health Challenges

The public health SIG was created to support the use of HIV cohort data to address public health challenges. Collaboration between national surveillance institutions and clinical cohorts can help address gaps in data availability; however, many countries have poor cross-communication or lack those channels for data sharing.

To reach the ambition of the 90-90-90 goals launched by the United Nations program on HIV/AIDS in 2014 [29], reliable estimates are needed on the size and the spread of the infection, the proportion on ART, and how many of those have viral suppression. Having provided proof of concept for using a smaller sample size to estimate the proportion of PLWH on ART and with viral suppression, the Public Health SIG will move on to conceptualize the method in an online tool. Subsequent studies will seek to validate this tool. The methodology could also play a role in assessing the TB and HCV disease burdens and treatment programs in locations where surveillance data is currently lacking.

The linked pilot PrEP and Resistance project (PrEPaRE; separate ethic approval: H-19043630, 13 November 2019) [30], which is also part of the Public Health SIG’s agenda, will investigate the frequency of prior PrEP use in individuals newly diagnosed with HIV. The project has initiated collaborations with more than 70 centers and, depending on the feasibility of reaching the enrollment numbers, follow-up studies will assess possible resistance patterns and correlate these to the drugs taken as part of PrEP.

The ability to quickly utilize the network within and around RESPOND is in part possible due to RESPOND’s widely used HICDEP-based data collection. This model also facilitates partnerships with other collaborations, as exemplified by the European Union’s horizon 2020 project Common Action Against HIV/TB/HCV Across the Regions of Europe (CARE) [31], where branches of CARE quickly adopted the RESPOND model to rapidly transfer local data on more than 4000 participants into one common data repository.

### 4.4. Limitations

As RESPOND is a non-randomized observational collaboration, the inherent limitations of possible missing data, selection bias, and unmeasured confounding apply for all analyses. To address the data quality and completeness, RESPOND employs a comprehensive and rigorous quality assurance system consisting of both automated and manual checks to ensure a high degree of data completeness and quality. In addition, the size of the study makes it possible to adjust for a wide range of possible confounding factors and perform adequately powered interaction and/or subgroup analyses. In addition, RESPOND will explore modern statistical methods such as causal inference, bioinformatics, and artificial intelligence.

At present RESPOND does not systematically collect data on resistance or HIV-subtype—both of which are subject to considerable heterogeneity in our study population [32,33], nor does the study collect data on use of therapeutics that have not yet been approved by the European Medicines Agency.

As the participating cohorts are based in Europe or Australia, the conclusion from RESPOND studies may not necessarily extend to PLWH from countries outside these regions and from resource-limited settings. Additionally, RESPOND includes only individuals from HIV cohorts able to comply with the RESPOND data collection principles and requirements.

## 5. Conclusions

In conclusion, RESPOND is a newly formed, flexible, and open consortium based on a well-established infrastructure and dedicated to research related to HIV and other infectious diseases. RESPOND has a geographically diverse study population; has a large data pool containing key HIV-related and study-specific information; employs a rigorous data quality program; and includes a collection of standardized, centrally validated clinical outcomes. These qualities place the consortium in a unique position to respond to a wide range of modern challenges for PLWH.

## Figures and Tables

**Table 1:** Overview of the RESPOND core variables collected by all the participating cohorts, and study-specific variables collected as part of the RESPOND substudies.

	RESPOND Core Variables	Study Specific Variables
**Demographics and Basic Information**	Date of birth	Ethnicity
	Sex	Country of origin
	Date first seen at department	Non-intravenous illicit drug use
	Date of first positive HIV1-Ab/Ag test	Intravenous drug use
	Height	
	Weigth	
	Baseline smoking status	
	Current smoking status	
	Risk of HIV acquisition of infection	
	Risk of HCV acquisition of infection	
	Risk of HBV acquisition of infection	
**Infection Related Laboratory Values**	HIV RNA	CD8 counts
	CD4 counts	HLA B*57:01
	HCV-antibodies	HCV genotype (and subtype)
	HCV RNA	Blood samples
	HBS Antigen	Tuberculosis diagnosis
	HBV DNA	Tuberculosis resistance
**Laboratory Values**	Creatinin	ALT
	Total cholesterol	AST
	HDL cholesterol	INR
	LDL cholesterol	Hemoglobin
	Triglycerides	Bilirubin
	HbA1C or blood glucose	Albumin
		Urine dipstick tests for proteinuria
Antiviral Treatment Treatment *	Nucleos(t)ide reverse transcriptase inhibitors	Hepatitis C treatment
	Non-nucleotide reverse transcriptase inhibitors	
	Protease inhibitors (cobicistat/ritonavir boosted or upboosted)	
	Integrase strand transfer inhibitors Entry inhibitors	
	Fusion inhibitors	
Non-Antiviral Treatment Treatment *	Anti-thrombotic drugs	Opioid substitution therapy
	Anti-hypertensive drugs	Anti-Tuberculosis treatment
	Anti-diabetic drugs	
	Lipid lowering drugs	
**Other Paraclinical Data**	Blood pressure	Bone Mass density: t-score
		Bone Mass density: z-score
		Bone Mass density area
		Liver trans elastography (fibroscan)
		Liver biopsy (metavir stage)
		Acoustic radiation force impulse (ARFI)
		HCC screening (Abdominal CT/MRI and ultrasound)
**Clinical Events**	Myocardial Infarctions **	Pregnancy
	Stroke **	
	Invasive cardiovascular procedures (coronary angioplasty/stenting, coronary bypass surgery, and carotid endarterectomi) **	
	Non-AIDS defining malignancies **	
	End-stage liver disease **	
	End-stage renal disease **	
	Fracture **	
	AIDS events (including AIDS defining cancers **)	
	Deaths ***	

*: Including start and stop dates and reasons discontinuations. **: A designated CRF must be filled out for events occurring after baseline. ***: A designated CRF for cause of death must be filled out for deaths occurring after baseline.

**Table 2:** Characteristics at the baseline after first data merger.

		*n*	%
**All**		26,258	100
**Age (Years)**	≤40	6488	24.9
	41–50	7909	30.4
	51–60	7811	30.0
	>60	3810	14.6
**Region**	Southern Europe (plus Argentina)	6933	26.6
	Western Europe	13,320	51.2
	Northern Europe (plus Australia)	2524	9.7
	Central Eastern Europe	1421	5.5
	Eastern Europe	1820	7.0
**Sex**	Male	19,329	74.3
	Female	6679	25.7
**Race**	White	18,834	72.4
	Black	4368	16.8
	Other/Unknown	2816	10.8
**HIV Acquisition Risk**	Men-sex-with-men	11,300	43.4
	Intraveneous drug use	3883	14.9
	Heterosexual	8931	34.3
	Other/Unknown	1904	7.3
Viral load (copies/mL) *	<200	24,073	89.5
	≥200	2185	10.5
	<LOD, where LOD> 200 cp/mL **	165	0.6
**ART naïve**	No	24,634	94.7
	Yes	1384	5.3
CD4 (cells/μL) ***	≤200	1410	5.4
	201–350	2689	10.3
	351–500	4429	17.0
	501–750	8758	33.7
	>750	8861	33.3
**CD4 Nadir (cells/μL)**	≤50	4268	16.4
	51–200	8233	31.6
	201–350	7813	30.0
	>350	5544	21.3
**Date of HIV diagnosis**	≤2011	20,723	79.6
	>2012	4494	17.3
	Unknown	801	3.1
**HBV status**	Negative	21,088	81.1
	Positive	1347	5.2
	Unknown	3583	13.8
**HCV status**	Negative	16,727	64.3
	Positive	6622	25.5
	Unknown	2669	10.3
	**Median**	**Interquartile Range**	
**Age (years)**	48	40–56	
**CD4 (cells/μL)**	621	438–830	
**CD4 Nadir (cells/μL)**	208	91–327	
**Baseline date (month/year)**	6/17	12/15–9/17	

* Viral load (VL) was unknown for 16 participants at the first data merger. ** LOD: Lower limit of detection. LOD was >200 copies/mL for the specific assay used for the participants’ latest measurement, with the results being a VL < than the specific LOD. *** CD4 was unknown for 71 participants at the first data merger.

## Appendix A

### Writing Group Members

Bastian Neesgaard ^1^: Amanda Mocroft ^2^, Lauren Greenberg ^2^, Jakob Friis Larsen ^1^, Gilles Wandeler ^3^, Robert Zangerle ^4^, Huldrych Günthard ^5^, Colette Smith ^6^, Stephane De Wit ^7^, Cristina Mussini ^8^, Antonella Castagna ^9^, Antonella D’Arminio Monforte ^10^, Jörg Janne Vehreschild ^11^, Jan-Christian Wasmuth ^12^, Christian Pradier ^13^, Nikoloz Chkhartishvili ^14^, Ferdinand W.N.M. Wit ^15^, Matthew Law ^16^, Anders Sönnerborg ^17^, Andreau Bruguera ^18^, Christoph Stephan ^19^, Vani Vannappagari ^20^, Richard Haubrich ^21^, Ole Kirk ^1^, Daria Podlekareva ^1^, Justyna Kowalska ^22^, Dorthe Raben ^1^, Juergen Rockstroh ^12^, Lars Peters ^1^, Roger Parades ^23^, Katharina Grabmeier-Pfistershammer ^24^, Alexandra Scherrer ^5^, Margaret Johnson ^6^, Coca Necsoi ^7^, Vanni Borghi ^8^, Camilla Muccini ^9^, Tengiz Tserstvadze ^14^, Kathy Petoumenos ^16^, Alain Volny-Anne ^25^, Jens Lundgren ^1^ and Lene Ryom ^1^

1. CHIP, Department of Infectious Diseases, Rigshospitalet, University of Copenhagen, Copenhagen, Denmark;
2. Centre for Clinical Research, Epidemiology, Modelling and Evaluation (CREME), Institute for Global Health, University College London, London, UK;
3. Department of Infectious Diseases, Bern University Hospital, University of Bern, Switzerland;
4. Austrian HIV Cohort Study (AHIVCOS), Medizinische Universität Innsbruck, Innsbruch, Austria;
5. Division of Infectious Diseases and Hospital Epidemiology, University Hospital Zurich, Zurich Switzerland;
6. Royal Free HIV Cohort, London, England;
7. Department of Infectious Diseases, St Pierre University Hospital Bruxelles, Brussels, Belgium;
8. Modena HIV Cohort, Università degli Studi di Modena, Modena, Italy;
9. San Raffaele Scientific Institute, Università Vita-Salute San Raffaele, Milano, Italy;
10. Italian Cohort Naive Antiretrovirals (ICONA), ASST Santi Paolo e Carlo, Milano, Italy;
11. University Hospital Cologne, Cologne, Germany;
12. Universitätsklinikum Bonn und Medizinische Fakultät, Bonn, Germany;
13. Nice HIV Cohort, Université Côte d’Azur et Centre Hospitalier Universitaire, Nice, France;
14. Georgian National AIDS Health Information System (AIDS HIS), Infectious Diseases, AIDS and Clinical Immunology Research Center, Tbilisi, Georgia;
15. AIDS Therapy Evaluation in The Netherlands (ATHENA) cohort, Stichting HIV Monitoring, Amsterdam, The Netherlands;
16. The Australian HIV Observational Database (AHOD), UNSW Sidney, Sydney, Austalia;
17. Swedish InfCare HIV Cohort, Karolinska University Hospital, Stockholm, Sweden;
18. PISCIS Cohort Study, Centre Estudis Epidemiologics de ITS i VIH de Catalunya (CEEISCAT), Badalona, Spain;
19. Frankfurt HIV Cohort Study, Johann Wolfgang Goethe-University Hospital, Frankfurt, Germany;
20. ViiV Healthcare, RTP, North Carolina, USA;
21. Gilead Sciences, Foster City, California;
22. Medical University of Warsaw, Poland;
23. Hosp. Universitari Germans Trias i Pujol, Badalona, Spain;
24. Austrian HIV Cohort Study (AHIVCOS), Medizinische Universität Wien, Vienna, Austria;
25. European AIDS Treatment Group (EATG)

## References

[1] The Data Collection on Adverse Events of Anti-HIV Drugs (DAD) Study Group (2003). Combination antiretroviral therapy and the risk of myocardial infarction. N. Engl. J. Med..

[2] Friis-Moller N., Weber R., Reiss P., Thiebaut R., Kirk O., d’Arminio Monforte A., Pradier C., Morfeldt L., Mateu S., Law M. (2003). Cardiovascular disease risk factors in HIV patients—Association with antiretroviral therapy. Results from the DAD study. AIDS.

[3] Laut K., Kirk O., Rockstroh J., Phillips A., Ledergerber B., Gatell J., Gazzard B., Horban A., Karpov I., Losso M. (2020). The EuroSIDA study: 25 years of scientific achievements. HIV Med..

[4] Chêne G., Phillips A., Costagliola D., Sterne J.A., Furrer H., Del Amo J., Mocroft A., d’Arminio Monforte A., Dabis F., Miro J.M. (2017). Cohort profile: Collaboration of observational HIV epidemiological research europe (COHERE) in EuroCoord. Int. J. Epidemiol..

[5] RESPOND Consortium Description v1.0 2019. https://chip.dk/Portals/0/files/RESPOND/RESPOND_Consortium-description_V1.0_2019MAY29.pdf?ver=20191–0-02-144627-317.

[6] RESPOND Governance and Procedures 2019. https://chip.dk/Portals/0/files/RESPOND/RESPOND%20governance%20and%20procedures_v6_2019SEP30.pdf?ver=2019-10-02-144419-230.

[7] WMA Declaration of Helsinki—Ethical Principles for Medical Research Involving Human Subjects. https://www.wma.net/policies-post/wma-declaration-of-helsinki-ethical-principles-for-medical-research-involving-human-subjects/.

[8] Commission Directive 2005/28/EC. https://eur-lex.europa.eu/eli/dir/2005/28/oj.

[9] RESPOND Project Proposal Template. https://chip.dk/Portals/0/files/RESPOND/RESPOND_study%20proposal%20template.doc?ver=2017-12-07-090247-223.

[10] About HICDEP. https://hicdep.org/Wiki/About-HICDEP.

[11] CHIP CC Standard Operating Procedurefor Data Transfer in RESPOND and EuroSIDA. vs 3.0. https://chip.dk/Portals/0/files/Eurosida/EuroSIDA/RESPOND_EuroSIDA_CARE_SOP_Electronic_Version3%200_2019_final.pdf?ver=2019-10-02-141500-817.

[12] About Project REDCap. https://projectredcap.org/about/.

[13] CHIP CC RESPOND Electronic Submission Tool (REST) User Guide vs 1.0. https://chip.dk/Portals/0/files/RESPOND/RESPOND_EuroSIDA_D45_Electronic_Submission_Tool_User_guide_Version1.pdf?ver=2017-12-07-091717-467.

[14] CHIP CC RESPOND Manual of Operations (MOOP). vs 1.6. https://chip.dk/Portals/0/files/RESPOND/RESPOND%20Manual%20of%20Operations%20MOOP__Version%201.6.pdf?ver=2019-11-05-124535-643.

[15] Greenberg L., Ryom L., Wandeler G., Grabmeier-Pfistershammer K., Öllinger A., Neesgaard B., Stephan C., Calmy A., Rauch A., Castagna A. (2020). Uptake and discontinuation of integrase inhibitors (INSTIs) in a large cohort setting. J. Acquir. Immune Defic. Syndr..

[16] Neesgaard B. Virologic and immunologic outcomes of integrase inhibitors (INSTIs). Proceedings of the Conference on Retrovirus and Oppotunistic Infections.

[17] Mocroft A. Virologic, immunologic and clinical outcomes in antiretroviral treatment (ART) naïve individuals in the RESPOND cohort collaboration (PE2/40). Proceedings of the 17th European AIDS Conference, EACS 2019.

[18] Greenberg L. Clinical outcomes of two drug regimens (2DRs) Vs. three drug regimens (3DRs) in HIV. In Proceedings of the Conference on Retrovirus and Opertunistic Infections.

[19] Raben D. (2019). A simple tool to evaluate the effectiveness of HIV care for settings with gaps in data availability. HIV Med..

[20] Lazarus J.V., Nielsen K.K. (2010). HIV and people over 50 years old in Europe. HIV Med..

[21] Gueler A., Moser A., Calmy A., Günthard H.F., Bernasconi E., Furrer H., Fux C.A., Battegay M., Cavassini M., Vernazza P. (2017). Life expectancy in HIV-positive persons in Switzerland: matched comparison with general population. AIDS.

[22] Althoff K.N., Smit M., Reiss P., Justice A.C. (2016). HIV and ageing: Improving quantity and quality of life. Curr. Opin. HIV AIDS.

[23] Guaraldi G., Orlando G., Zona S., Menozzi M., Carli F., Garlassi E., Roverato A., Palella F. (2011). Premature age-related comorbidities among HIV-infected persons compared with the general population. Clin. Infect. Dis..

[24] Holtzman C., Armon C., Tedaldi E., Chmiel J.S., Buchacz K., Wood K., Brooks J.T., HOPS Investigators (2013). Polypharmacy and risk of antiretroviral drug interactions among the aging HIV-infected population. J. Gen. Intern. Med..

[25] Troya J., Bascunana J. (2016). Safety and tolerability: Current challenges to antiretroviral therapy for the long-term management of HIV infection. AIDS Rev..

[26] World Health Organization, Fact Sheets; Top 10 Causes of Death. https://www.who.int/news-room/fact-sheets/detail/the-top-10-causes-of-death2018.

[27] World Health Organization, Fact Sheets; Tuberculosis. https://www.who.int/news-room/fact-sheets/detail/tuberculosis2020.

[28] Podlekareva D.N., Efsen A.M., Schultze A., Post F.A., Skrahina A.M., Panteleev A., Furrer H., Robert F.M., Losso M.H., Toibaro J. (2016). Tuberculosis-related mortality in people living with HIV in Europe and Latin America: an international cohort study. Lancet HIV.

[29] (UNAIDS) UNPoHA 90-90-90 An Ambitious Treatment Target to Help end the AIDS Epidemic United Nations Programme on HIV/AIDS (UNAIDS). http://files.unaids.org/en/media/unaids/contentassets/documents/unaidspublication/2014/90-90-90_en.pdf2014.

[30] The PrePaREstudy. https://chip.dk/Studies/PrEPaRe.

[31] (2019). CARE East Cohort Protocol (A Study in the RESPOND Consortium) Version 1.0. https://chip.dk/Portals/0/files/CARE/CARE-East_Cohort_Protocol_V1.0_2019AUG16.pdf?ver=2019-12-03-130.

[32] Schultze A., Phillips A.N., Paredes R., Battegay M., Rockstroh J.K., Machala L., Tomazic J., Girard P.M., Januskevica I., Gronborg-Laut K. (2015). HIV resistance testing and detected drug resistance in Europe. AIDS.

[33] 2Bbosa N., Kaleebu P., Ssemwanga D. (2019). HIV subtype diversity worldwide. Curr. Opin. HIV AIDS.

